# Multimodal platform for ITN efficacy: Surface chemistry, bioavailability, and mosquito behavior

**DOI:** 10.1126/sciadv.aeb2023

**Published:** 2026-04-08

**Authors:** Hanafy M. Ismail, Nga Tsing Tang, Jeff Jones, Kyle Walker, Jonathan Thornton, Mark Paine, Stephan Karl, Lisa Reimer, Rasmita Raval

**Affiliations:** ^1^ Liverpool School of Tropical Medicine, Pembroke Place, Liverpool L35QA, UK.; ^2^ Open Innovation Hub for Antimicrobial Surfaces and Surface Science Research Centre, Department of Chemistry, University of Liverpool, Liverpool L69 7ZD, UK.; ^3^ PNG Institute of Medical Research, Madang, Madang Province, Papua New Guinea, Australian Institute of Tropical Health and Medicine, James Cook University, Smithfield, Queensland, Australia.; ^4^ Entomology Branch, Division of Parasitic Diseases and Malaria, National Center for Emerging and Zoonotic Infectious Diseases, Centers for Disease Control and Prevention, Atlanta, GA USA.

## Abstract

Insecticide-treated nets (ITNs) are crucial for malaria control, but their efficacy is compromised by rising mosquito resistance. To better understand ITN effectiveness, we present a multidisciplinary framework through a case study examining the removal of per- and polyfluoroalkyl substances (PFAS) from ITN coatings and its impact on malaria vectors in East and West Africa. Our results show that PFAS-free pyrethroid nets exhibit reduced bio-efficacy against resistant malaria vectors compared with PFAS-based nets, despite both meeting deltamethrin specifications. Surface characterization reveals that PFAS stabilizes smaller, noncrystalline deltamethrin particulates enhancing bioavailability, while PFAS-free coatings promote particulate aggregation with an increased population of crystalline deltamethrin. Behavioral assays suggest that PFAS-free formulations reduce mosquito contact time and insecticide uptake, with resistant strains showing decreased irritancy and knockdown. These findings highlight the trade-offs of PFAS removal and stress the need for a multidisciplinary approach combining advanced chemical analytics and behavioral assessments to optimize ITNs for effective malaria control while considering environmental sustainability.

## Teaser

Removing PFAS from ITNs compromises insecticide bioavailability and effectiveness, particularly against resistant malaria vectors.

## INTRODUCTION

Malaria, a parasitic disease transmitted between humans by mosquitoes, remains a substantial global health challenge, with more than 200 million cases reported each year (*1*). Insecticide-treated nets (ITNs) have been a key preventive tool, offering both a physical barrier and insecticidal efficacy to kill mosquitoes and reduce parasite transmission. Their widespread use in Africa has notably reduced the malaria burden (*2*). However, these gains are threatened by multiple factors, ranging from chemical and physical alterations of the insecticide that affect bio-efficacy to rising insecticide resistance (*3*). Here, we show that a multidisciplinary approach that integrates advanced physical and chemical analytics with behavior studies that map mosquito-ITN interactions provides a powerful route to understanding bio-efficacy in both resistant and nonresistant strains and provides a crucial framework to drive sustained efficacy (*4*) and innovation (*3*).

Papua New Guinea (PNG), which has the highest malaria transmission rates outside Africa, serves as a notable case study of the achievements (*5*) and challenges related to ITNs (*6*). The introduction of deltamethrin ITNs in PNG resulted in a large drop in malaria prevalence, from 16% in 2008 to just 1% in 2014 (*5*, *7*). Furthermore, it is remarkable that, after nearly 2 decades of ITN mass distributions in PNG, there is no indication of pyrethroid resistance developing in the local anopheline populations (*8*). However, since 2015, malaria has resurged sharply in PNG, coinciding with reduced ITN efficacy (*6*). This decline has been attributed to formulation changes in PermaNet 2.0 implemented after 2012, specifically the transition from per- and polyfluoroalkyl substances (PFAS)–based coatings to non-PFAS alternatives, likely affecting insecticide retention and bioavailability (*9*). This change reflects growing global concerns regarding PFAS due to environmental and health risks (*10*, *11*). While they have not been widely phased out, their removal from ITN coatings may have unintended public health consequences, highlighting the need to balance safety and efficacy.

PermaNet 2.0 (75 Denier) is treated postweaving with 54 mg ± 25% deltamethrin, offering strong protection against susceptible mosquito populations (*12*, *13*). However, the growing spread of pyrethroid resistance has diminished the effectiveness of pyrethroid-only nets like PermaNet 2.0 (*4*), leading to the development of dual active ingredient (AI) ITNs such as Royal Guard, Interceptor G2, and PermaNet Dual to support continued malaria control. Mosquitoes have evolved resistance mechanisms that diminish pyrethroid effectiveness through knockdown resistant (*kdr*) mutations, which alter the voltage-gated sodium channel (VGSC) target site, and metabolic resistance, where detoxifying enzymes (cytochrome P450s, glutathione *S*-transferases, and esterases) accelerate insecticide breakdown, thus reducing efficacy (*14*). Additionally, overexpression of sensory appendage protein (SAP2), which is enriched in mosquito legs, has shown to confer pyrethroid resistance via high-affinity binding with pyrethroids (*15*). Moreover, behavioral changes, including altered feeding times or locations, may allow mosquitoes to avoid contact with ITNs (*16*, *17*). To study these behavioral adaptations, researchers recently used high-resolution and frame-rate imaging of mosquito-ITN interaction to examine mosquito interactions with pyrethroid-only ITNs, next-generation (dual-action) nets, and aged versus new nets (*18*–*20*). Collectively, these physiological and behavioral resistance mechanisms highlight the urgent need to reform and adapt ITN quality assurance (QA) systems to meet evolving resistance challenges.

**Fig. 1.**
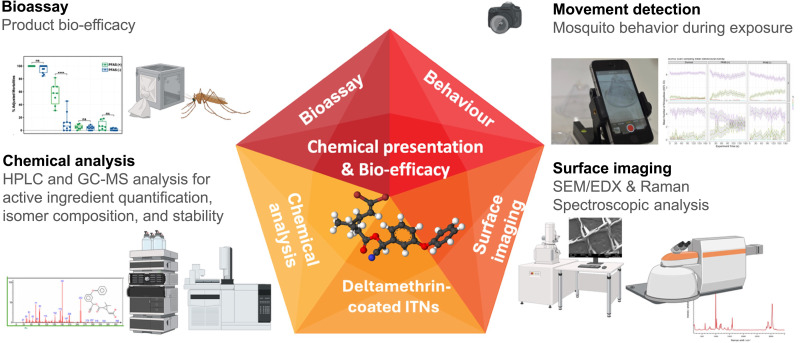
Integrated analytical framework for evaluating ITNs, focusing on chemical presentation and bio-efficacy. The schematic illustrates four interconnected components that together provide a comprehensive assessment of ITN performance. Bioassays are used to determine product bio-efficacy through standardized mosquito exposure protocols. Chemical analysis via HPLC and GC-MS enables quantification of AIs, identification of isomeric forms, and assessment of chemical stability. Surface imaging, using scanning electron microscopy (SEM)/energy-dispersive x-ray (EDX) and Raman spectroscopy, provides insight into the spatial distribution and molecular characteristics of insecticide deposition on net fibers. Video analysis technologies are used to monitor mosquito behavior during exposure, offering dynamic data on contact duration, avoidance, and sublethal responses. These components converge to define the chemical presentation of the insecticide and its bio-efficacy, forming a core metric for comparing ITN performance across products and resistance profiles.

Current QA protocols rely on analytical techniques such as high-performance liquid chromatography (HPLC) and gas chromatography (GC) to measure the total deltamethrin content, along with bioassays to evaluate mosquito mortality. However, these methods overlook critical factors such as the surface presentation and availability of the AI (deltamethrin) and its interaction with resistance mechanisms. To bridge these gaps, we created a multidisciplinary analytical framework summarized in Fig. 1, incorporating HPLC for insecticide quantification, GC–mass spectrometry (MS) for chemical composition analysis, scanning electron microscopy (SEM)/energy-dispersive x-ray (EDX) and Raman confocal microscopy for imaging surface presentation and assessing local chemistries, video cone assays to evaluate mosquito behavior and mortality, and high-resolution, high-frame-rate imaging to study mosquito-ITN interactions. This approach aimed to correlate changes in insecticide content and its surface presentation with ITN efficacy against *Anopheles* mosquitoes, including resistant and susceptible colonies. Two batches of PermaNet 2.0 from PNG, produced with PFAS in 2012 [PFAS (+) net] and without PFAS in 2019 [PFAS (−) net], were analyzed through the multidisciplinary framework to explore whether changes to the formulation could have an impact on the surface presentation of the insecticide. By combining advanced analytical techniques with behavioral assessments, we established a comprehensive platform to support improved ITN design, through a better understanding of bio-efficacy, and to allow sustained progress in malaria control in the face of rising insecticide resistance.

## RESULTS

### Bioassay evaluation of PFAS (+) and PFAS (−) sample efficacy across mosquito strains

**Fig. 2.**
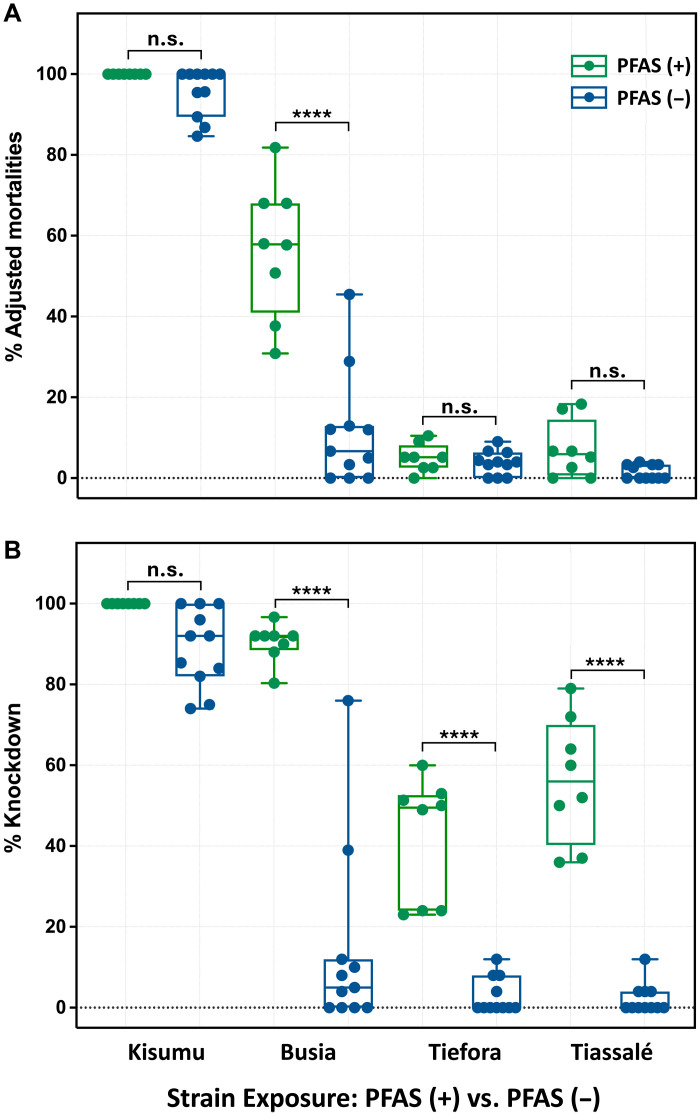
Declining formulations efficacy of PFAS (+) and PFAS (−) samples against *Anopheles* mosquito strains. (**A**) Adjusted mortality (%) of Kisumu, Busia, and Tiassalé (*An. gambiae*) and Tiefora (*An. coluzzii*) strains exposed to PFAS (+) and PFAS (−) samples from 2012 [green; PFAS (+) net] and 2019 [blue; PFAS (−) net]. Boxplots display median, interquartile range, and individual data points from 8 PFAS (+) net and 11 PFAS (−) net swatches, highlighting significantly lower mortality in PFAS (−) net for Busia (****P < 0.0001), while no significant differences were observed for Kisumu, Tiefora, or Tiassalé [not significant (n.s.)], based on one-way analysis of variance (ANOVA) with Tukey’s post hoc test. Each data point represents the average of five replicate assays per net swatch, totaling 25 mosquitoes per test (file S1). Negative controls using untreated net swatches were conducted simultaneously, as detailed in the methodology. All assays were performed under controlled conditions at 27° ± 2°C and 80 ± 20% relative humidity. (**B**) Knockdown (KD; %) of the same strains exposed to PFAS (+) net (2012) and PFAS (−) net (2019) ITNs revealing significantly lower KD rates for Busia, Tiefora, and Tiassalé strains in the 2019 batch (****P < 0.0001), while Kisumu remained unaffected, suggesting a reduction in insecticidal efficacy over time, likely influenced by evolving resistance profiles in these malaria vector populations.

Bioassay evaluation of PFAS (+) and PFAS (−) net samples across mosquito strains, with varying resistance levels, revealed distinct efficacy patterns in both 24-hour mortality and 1-hour knockdown (KD) responses (Fig. 2). Both net batches were highly effective against the susceptible Kisumu strain, achieving with mortality rates of 100 and 96%, respectively (Fig. 2A). The most notable differences between net types were observed in the partially resistant Busia strain (Uganda), where mortality plummeted from 56 to 11% at *P* < 0.0001 (Fig. 2A). This disparity may reflect differences in insecticide bioavailability in the context of the high prevalence (91.7 to 98.9%) of the *Vgsc-995S* allele, a key East African kdr marker associated with deltamethrin resistance (*21*). Among the highly resistant West African strains Tiassalé (Côte d’Ivoire) and Tiefora (Burkina Faso), there is a consistently poor response to both net types, with low mortality rates (Tiassalé: 7% versus 1.4%; Tiefora: 5.0% versus 3.7% for PFAS (+) and PFAS (−), respectively; Fig. 2A). Resistance in these strains is primarily due to metabolic detoxification mechanisms, alongside *kdr-*associated reduction in pyrethroid sensitivity due to altered binding at the VGSC. The *Vgsc*-*995F* allele (formerly *Vgsc*-1014F) is highly prevalent in Tiassalé (83 to 79%) (*22*, *23*) but rare in Tiefora (6%) (*24*), suggesting a stronger contribution of metabolic resistance in Tiefora.

Both East (*Vgsc*-995S) and West (*Vgsc*-995F) *kdr* alleles reduce pyrethroid efficacy by impairing insecticide binding to VGSC (*25*–*28*). Despite metabolic resistance variations allowing some mosquitoes to recover or die, KD mortality remains a reliable indicator of insecticide bioavailability (*27*). A strong correlation was observed between *kdr* allele frequency and KD after 1-hour exposure, highlighting subtle differences between the two ITN batches (Fig. 2B). The reduction in KD percentage varied among strains and formulations, with Busia (*Vgsc*-995S allele frequency, 91.7 to 98.9%) experiencing the most significant decline from 92% [PFAS (+) net] to just 14% [PFAS (−) net] (*P* < 0.0001; Fig. 2B). Tiassalé (*Vgsc*-995F allele frequency, 79.3%) also experienced a sharp decline, from 56.25% KD for PFAS (+) net to 2.18% for PFAS (−) net, while Tiefora (*Vgsc*-995F allele frequency, 6%) saw a milder but still substantial reduction, from 41.79 to 2.91%. In contrast, the Kisumu strain, which lacks major resistance mechanisms, showed only a minor but nonsignificant decline, 100% KD [PFAS (+) net] versus 89% [PFAS (−) net] (*P* > 0.05; Fig. 2B). Collectively, these findings underscore the poorer performance of PFAS (−) net compared with that of PFAS (+) net against resistant mosquitoes.

### Chemical analysis of PFAS (+) and PFAS (−) net samples: Insights into efficacy variations

**Fig. 3.**
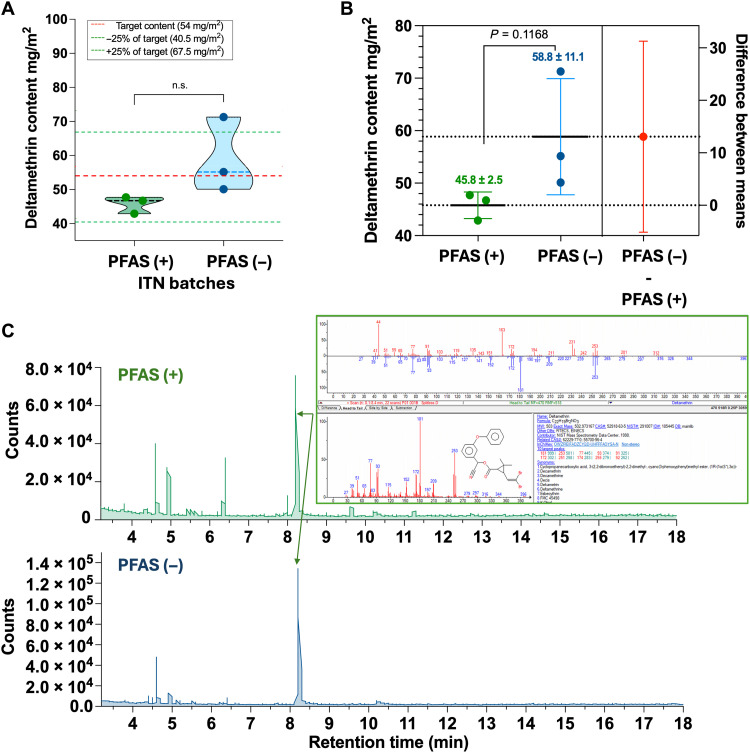
Deltamethrin content analysis in samples manufactured in PFAS (+) net and PFAS (−) net. (**A**) Violin plot showing the quantified deltamethrin content [milligrams per square meter (mg/m^2^)] for PFAS (+) and PFAS (−) ITNs analyzed from five panels in triplicates. The horizontal dashed lines represent the target deltamethrin content (red, 54 mg/m^2^) and ±25% range (green, 40.5 to 67.5 mg/m^2^). Data points indicate individual samples, and the *P* value (*P* = 0.1168) reflects the result of an independent *t* test comparing mean. (**B**) Error-bar plot displaying the mean deltamethrin content (±SD) for PNG2012 (green, 45.8 ± 2.5 mg/m^2^) and PNG2019 (blue, 58.8 ± 11.1 mg/m^2^). The red point indicates the difference between the means, with the dashed horizontal line at zero for reference. (**C**) MS chromatograms illustrating the detected deltamethrin signal for PNG2012 (green) and PNG2019 (blue) ITNs, with the molecular weight of deltamethrin (*M*_w_, 503) indicated. Insets show mass spectra and the chemical structure of deltamethrin, highlighting key fragments.

Building on the bioassay results, we analyzed the same PermaNet 2.0 net samples to identify chemical factors influencing the observed efficacy differences. First, HPLC was used to measure deltamethrin content, to assess whether both batches met the manufacturer’s target of 54 mg/m^2^, within an acceptable range of 40.5 to 67.5 mg/m^2^ (Fig. 3A). Both the PFAS (+) and PFAS (−) nets contained deltamethrin within this range, with mean values near the target dose. The difference of ~14 mg/m^2^ between batches was not statistically significant (*P* = 0.1168; Fig. 3B). Further confirmatory analysis using GC–tandem MS verified the presence of the biologically active (1*R*,3*R*)-configured deltamethrin stereoisomer [(*S*)-α-cyano-3-phenoxybenzyl (1*R*,3*R*)-3-(2,2-dibromovinyl)-2,2-dimethylcyclopropane-1-carboxylate] in both PFAS (+) and PFAS (−) samples, with no signs of isomerization or chemical degradation. This was consistently observed at a retention time of 8.2 min (Fig. 3C).

Overall, the PFAS (+) net swatches demonstrated a more consistent deltamethrin application, with an average content of 45.8 mg/m^2^ and minimal variability (±2.5 mg/m^2^). All samples remained within the acceptable range, supported by a low relative SD (RSD) of 5.54%, suggesting an even distribution of the deltamethrin across the 2012 net batch (table S1). In contrast, the PFAS (−) net sample exhibited higher deltamethrin peak counts on GC-MS, consistent with HPLC results showing that the 2019 net batch had a higher average deltamethrin content of 58.8 mg/m^2^. However, the deltamethrin content displayed distinct variability in this net in comparison to PFAS (+) net, ranging from 50.1 to 71.3 mg/m^2^, with a RSD of 18.81% (table S1). The reduced mortality observed with PFAS (−) net samples cannot be attributed to its deltamethrin content as these ITNs met target levels and had a higher average concentration than PFAS (+) net samples. Therefore, we investigated whether the surface presentation of the AI could contribute to the reduced insecticidal efficacy of PFAS (−) net, particularly against resistant strains such as Busia and Tiassalé.

### Surface analysis of deltamethrin presentation in PFAS (+) and PFAS (−) samples

#### Surface morphology analysis

**Fig. 4.**
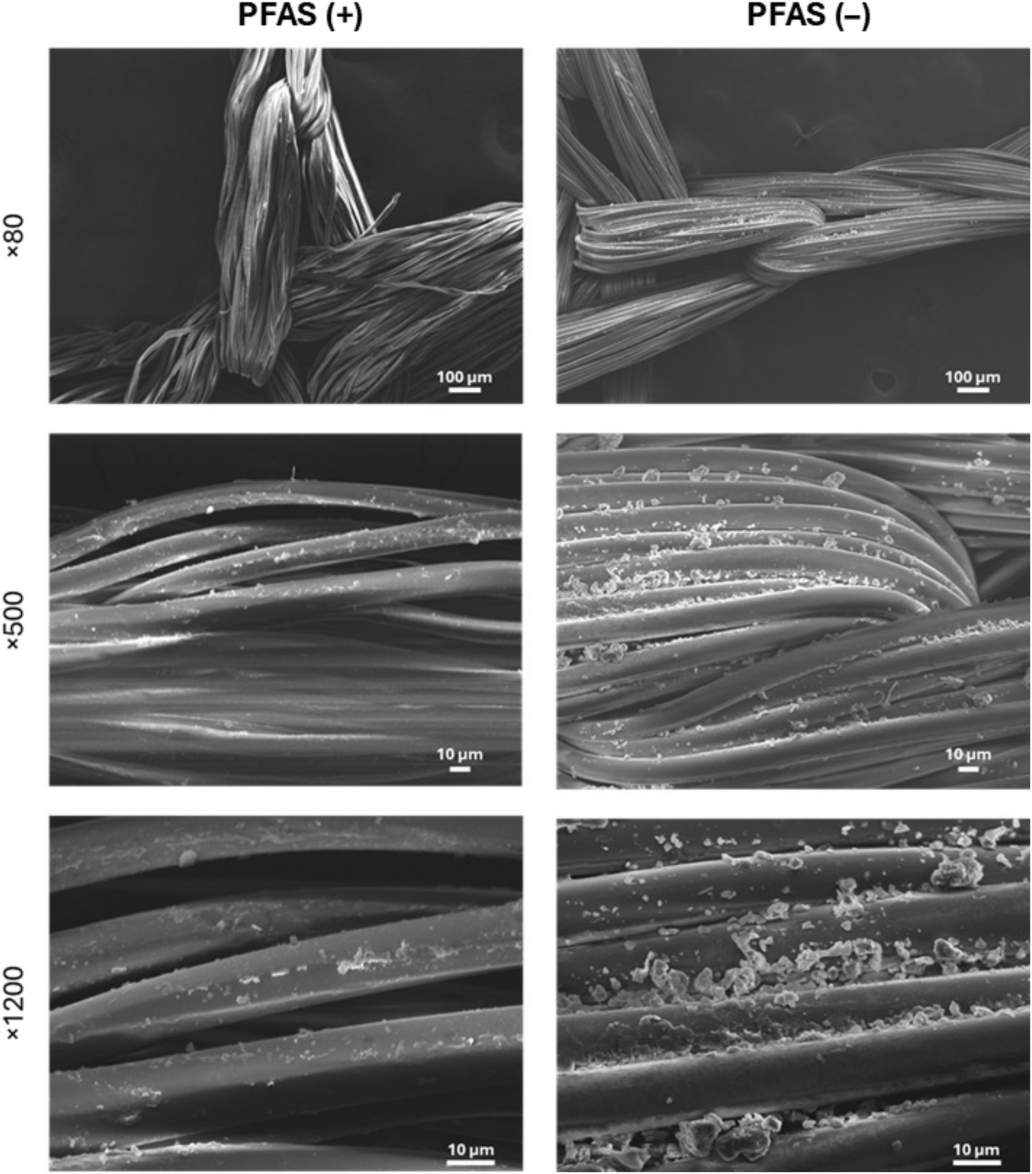
Imaging surface presentation of deltamethrin on PFAS (+) net and PFAS (−) net using SEM. SEM images of samples from PFAS (+) net (left column) and PFAS (−) net (right column) captured at magnifications of ×80, ×500, and ×1200. The images reveal notable differences in the density and size of deltamethrin particulates on the fibers, with increased particulate presence observed in the PFAS (−) net. These variations in deltamethrin presentation may affect the bioavailability and efficacy of the nets. Scale bars, 100 μm (×80) and 10 μm (×500 and ×1200).

SEM analysis of PFAS (+) and PFAS (−) nets from 2012 and 2019, respectively, revealed pronounced differences in the surface presentation of deltamethrin particulates (Fig. 4). The PFAS (−) net exhibited a notably higher density of particulates at all magnifications compared with the PFAS (+) net, along with larger particulates that possess a wider size distribution (Fig. 4). These morphological differences may explain the paradoxical observation of lower mosquito mortality rates associated with the PFAS (−) net, despite their higher insecticide content as measured by HPLC. The formulation and coating method changes in PermaNet 2.0 were primarily driven by the industry-wide shift away from PFAS-based binders, which were discontinued due to regulatory concerns (*29*). These modifications aimed to maintain compliance with global standards while ensuring the insecticides’ continued bioavailability and efficacy. However, the differences in size and size distributions of deltamethrin particulates raise issues about altered release or depletion dynamics, potentially affecting both bioavailability and wash resistance, which would affect long-term performance (*9*).

#### Elemental analysis reveals compositional variations in PFAS (+) and PFAS (−) samples

**Fig. 5.**
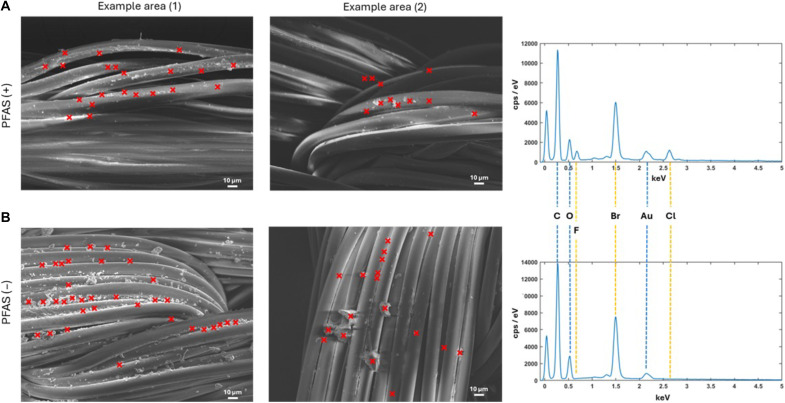
Localized chemical composition of deltamethrin particulates on PFAS (+) and PFAS (−) nets analyzed by SEM and EDX. (**A**) Top row, PFAS (+) net: SEM images at ×500 magnification from two example areas on the PFAS (+) net surface. Area 1 represents the same PFAS (+) area shown in Fig. 4 at ×500 magnification. Corresponding EDX spectra were obtained at specific particulate locations marked with crosses. The representative EDX spectrum from PFAS (+) net reveals the presence of bromine (Br), fluorine (F), and chlorine (Cl), confirming deltamethrin and specific PFAS formulation. (**B**) Bottom row, PFAS (−) net: SEM image at ×500 magnification from two example areas on the PFAS (−) net surface. Area 1 represents the same PFAS (−) sample area shown in Fig. 4 at ×500 magnification. Corresponding EDX spectra were obtained at the locations marked with crosses. The representative EDX spectrum of PFAS (−) net shows the presence of bromine (Br) but no fluorine (F) or chlorine (Cl), indicating a change in the formulation used in 2019. cps, counts per second.

Given the distinctly different deltamethrin particulate morphology and size distributions observed between PFAS (+) and PFAS (−) net samples (Fig. 4), combined SEM and EDX analysis was performed to further characterize the particulates and their elemental composition (Fig. 5). This approach enabled visualization of individual particulates via SEM and the identification of their elemental makeup using EDX. As seen in Fig. 4, SEM images at ×500 magnification highlighted distinct differences between the two formulations. PFAS (+) net featured smaller, more uniformly size-distributed particulates on the fiber surfaces, while PFAS (−) net showed larger, denser, and more irregularly size-distributed particulates. EDX spectra confirmed the presence of bromine (Br), a key element in deltamethrin, in the particulates observed for ITN formulations, verifying the presence of insecticide. However, PFAS (+) nets uniquely contained fluorine (F), which was absent in PFAS (−) net, indicating the shift in formulation (*9*). The absence of fluorine in PFAS (−) net aligns with findings by Bubun *et al.* (*9*), linking reduced ITN effectiveness post-2012 to the shift from PFAS-based coatings to non-PFAS alternatives (*9*). These morphological differences in the particulates alongside compositional changes, arising from the absence of the fluorine-containing binder in PFAS (−) net, reveal additional potential contributors to reduced efficacy against resistant mosquitoes. For example, the larger particulate sizes observed in PFAS (−) net compared with PFAS (+) net may be expected to affect deltamethrin insecticidal efficacy, due to reduced surface-to-volume ratios. In addition, potential changes in crystal form need to be considered as discussed in the next section.

#### Molecular analysis of surface particulates using Raman microscopy

**Fig. 6.**
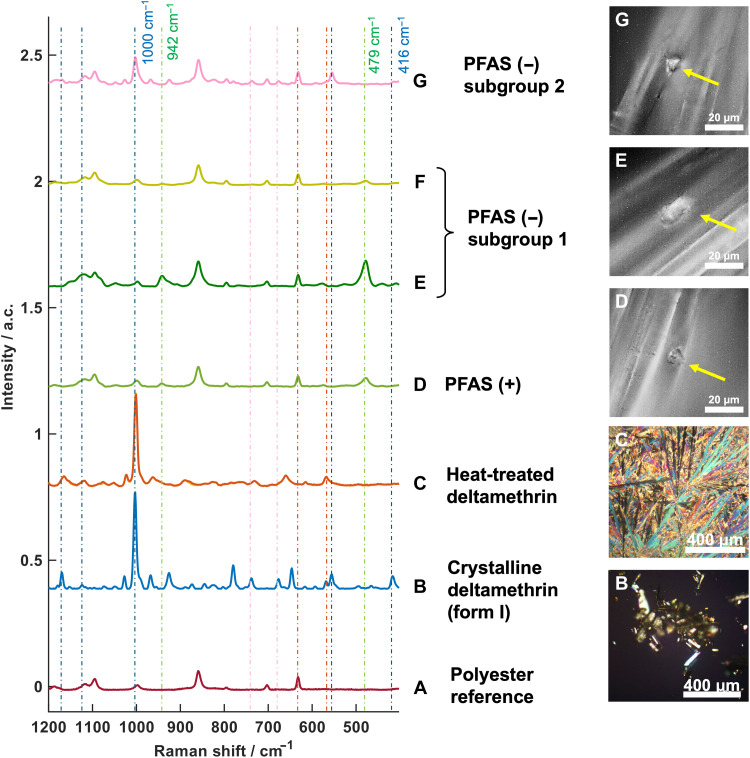
Vibrational Raman spectra and optical images of deltamethrin particulates on PFAS (+) and PFAS (−). (**A**) Raman spectrum of a polyester reference. (**B**) Raman spectrum of crystalline deltamethrin (form I) and its corresponding polarized light microscopy image. (**C**) Raman spectrum of heat-treated deltamethrin and its polarized light microscopy image. (**D**) Raman spectrum of particulates from the PFAS (+) net and a representative optical image. (**E** and **F**) Raman spectra of subgroup 1 particulates from the PFAS (−) net and associated optical images. (**G**) Raman spectrum of representative subgroup 2 particulates from the PFAS (−) net and associated optical image. Yellow arrows in the optical images indicate the analyzed particulates. a.c., aberrative counts.

It has been shown that deltamethrin’s transition from stable form I to metastable form II when heated and recooled substantially enhances insecticidal activity and uptake and overcomes resistance (*30*). Therefore, further analysis of the particulates found on the net surfaces was undertaken with confocal Raman microscopy, a vibrational spectroscopic technique that can provide information on chemical composition, phase, morphology, and crystallinity at the local level. For this work, reference Raman spectra were acquired from the PFAS (+) and PFAS (−) samples after deltamethrin was chemically removed through multiple methanol washes to representing the polyester net fibber composition (Fig. 6A) and from commercially sourced neat crystalline deltamethrin form I (Fig. 6B). Additionally, reference Raman spectra were obtained after heating form I to 110°C to induce melting, followed by recooling to room temperature (Fig. 6C). Polarized light microscopy images in Fig. 6 reveal the appearance of bright fibrils postheating, indicative of the transition from form I to form II, as described by Yang *et al.* (*31*). While the Raman spectra of the unheated and heated forms in the region at 1200 to 400 cm^−1^ are largely similar, the heated form exhibits notable peak shifts, intensity loss, and band broadening, as detailed in fig. S1.

Raman microscopy spectra were then acquired from individual particulates on the surfaces of PFAS (+) and PFAS (−) nets, with multiple particulates analyzed for each net type. The results reveal that PFAS (+) net contains only one type of particulate, producing a consistent Raman spectrum (Fig. 6D and fig. S2). Notably, these particulates do not show the major characteristic deltamethrin features observed in either of the reference spectra for either crystalline form I or form II. Instead, the spectrum is dominated by polyester fiber peaks, with additional bands at 942 and 479 cm^−1^. Specifically, the strongest crystalline deltamethrin peak at 1000 cm^−1^, arising from the νring + δ (C-C) in-plane mode (*32*), is absent, with only the polyester fiber contributing to the Raman signal in this region. This observation contrasts with HPLC, GC-MS, and EDX data confirming the presence of deltamethrin on PFAS (+) net, as well as bioassays demonstrating its efficacy. This discrepancy suggests a unique physical or chemical state of the deltamethrin in PFAS (+) net, potentially involving a loss of crystalline order within the particulates. Crystalline forms typically exhibit sharp, intense Raman peaks due to long-range vibrational coupling and defined local chemical environments (*33*, *34*). In contrast, amorphous solids or molecular dispersions, lacking long-range order and exhibiting heterogeneous chemical environments, will often display broadened spectral lines that can span hundreds of wave numbers, making them difficult to discern against the baseline (*33*, *34*). The absence of the sharp peak at 1000 cm^−1^ in PFAS (+) net indicates that deltamethrin may exist in an amorphous or molecularly dispersed form, instead of its crystalline state, which could explain the significant loss of intensity of Raman peaks, making them imperceptible in a confocal Raman spectrometer (*35*).

Raman microscopy spectra at the individual particulates level were also collected from the PFAS (−) net (Fig. 6, E to G, and fig. S2). Clear differences emerged between the two types of ITNs with Raman spectra of the PFAS (−) net particulates separating into two subgroups. Subgroup 1 shows Raman features (Fig. 6, E and F), which are similar to those exhibited by the PFAS (+) net particulates (Fig. 6D), that is, the particulates do not display signals indicative of crystalline deltamethrin. In contrast, subgroup 2 particulates show spectroscopic features (Fig. 6G) that resemble crystalline deltamethrin form I (Fig. 6B). These differences in deltamethrin form could have arisen from the change in formulation and the manufacturing process used in the PFAS (+) and PFAS (−) nets. The nature of deltamethrin in an active formulated particulate would substantially affect its bio-efficacy. It has been shown that crystalline deltamethrin form II has a higher bio-efficacy compared with crystalline deltamethrin form I (*31*). The lethality of crystalline insecticides generally decreases as the crystalline form becomes more stable (*36*), effectivity increasing the barrier of dissociating the molecule from its crystalline form before uptake into the tarsi. We propose that the noncrystalline or molecularly dispersed forms of deltamethrin particulates may be even more bio-effective than the crystal forms I and II because the deltamethrin is no longer held within a stable crystalline structure and hence more bioavailable. It would appear that the PFAS (+) net formulation stabilizes a noncrystalline form of deltamethrin, while the PFAS (−) net formulation presents two different populations of the active: the more toxic form (noncrystalline) alongside the less effective one in the stable form I crystal.

### Behavioral responses of *Anopheles gambiae* to PFAS (+) and PFAS (−) samples

Given the compositional and structural differences between the 2012 and 2019 batches of PFAS (+) and PFAS (−) samples, we investigated how these chemical variations influence mosquito behavior by assessing their performance against the pyrethroid-resistant Tiassalé strain and the susceptible Kisumu strain. Tiassalé was selected for detailed behavioral analysis on the basis of our initial bioassay data, which demonstrated limited efficacy (mortality) of both net batches against this resistant population, with the PFAS (+) net showing notably higher KD rates compared with the PFAS (−) net formulation (Fig. 2). The Kisumu strain, serving as a susceptible control, provided a baseline for comparing behavioral responses to the treated nets, offering insight into strain-specific interactions with these formulations.

#### Total movement events

**Fig. 7.**
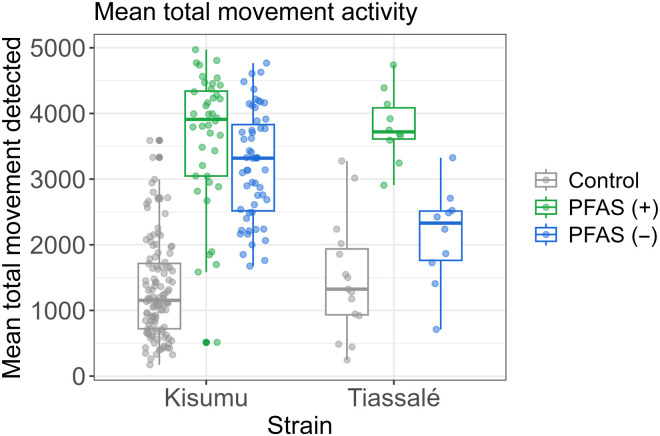
Mean total mosquito activity distribution across PFAS (+) and PFAS (−) samples and strains. Boxplots depicting the distribution of mean total mosquito activity detected using the Video Cone Test Analysis (ViCTA) method for two mosquito strains Kisumu and Tiassalé, exposed to three net types: control (red), PFAS (+) net (green), and PFAS (−) net (blue). Each point represents the total count of detected movements in an individual cone test. The boxplots summarize the median and interquartile range for each net type. The chart highlights differences in mosquito activity levels across the net types and between the two mosquito strains.

Using Video Cone Test Analysis (ViCTA) software, mosquito activity was evaluated in the cone for two strains, Kisumu and Tiassalé, exposed to a negative control, PFAS (+) and PFAS (−) nets (Fig. 7). Kisumu strain exhibited highest total activity on PFAS (+) net (mean, 3628.30; 95% CI, 3303.94 to 3952.66) but activity declined on PFAS (−) net (mean, 3211.65; 95% CI, 2987.19 to 3436.12). Lowest activity was observed with the control net (mean, 1324.42; 95% CI, 1190.18 to 1458.66). Elevated activity on treated nets is caused by excito-repellency due to deltamethrin sensitivity (Fig. 7), resembling the pattern of KD observations in Fig. 2. In contrast, Tiassalé strain showed slightly higher activity than Kisumu on PFAS (+) net (mean, 3804.00; 95% CI, 3424.00 to 4184.00) but a more notable reduction in activity on PFAS (−) net (mean, 2141.60; 95% CI, 1613.29 to 2669.91). Control net activity was also low for Tiassalé (mean, 1484.60; 95% CI, 995.02 to 1974.18) but slightly higher than Kisumu, indicating an innately higher level of movement for this strain. A descriptive data summary is shown in table S2. No significant difference in variance was found across groups (Levene’s test, df = 5, *F* = 1.2468, *P* = 0.2878). Two-sample independent Welch’s *t* tests were performed to compare total movement activity detected in video cone tests (VCTs).

For the susceptible strain Kisumu, there was a statistically significant difference in total movement between PFAS (+) net (mean = 3628.3, SD = 1014.209) and PFAS (−) net (mean = 3211.655, SD = 830.311) (*t* = 2.130, df = 73.649, *P* = 0.036). The effect size of this difference according to Cohen’s *D* measure was *d* = 0.457 (CI, 0.039 to 0.875), indicating a small effect. For the resistant strain Tiassalé, a statistically significant difference in total movement was also found between PFAS (+) net (mean = 3804, SD = 531.208) and PFAS (−) net (mean = 2141.6, SD = 738.529) (*t* = 5.778, df = 16.346, *P* < 0.001). The effect size of this difference was *d* = 2.584 (CI, 1.312 to 3.857), indicating a large effect. A two-way analysis of variance (ANOVA) was performed to analyze the effect of strain and net distribution type on the total detected mosquito movement. The numerical dependent variable was total movement. The categorical independent variables were strain (Kisumu or Tiassalé) and net distribution [PFAS (+) net, PFAS (−) net, and control nets]. Residuals of the ANOVA model were manually analyzed with a QQ normal plot and assessed using the Shapiro-Wilk test giving a result of (*W* = 0.99154, *P* = 0.1597), indicating that the residuals were normally distributed.

Simple main effects analysis demonstrated that mosquito strain was not significant (95%) as a main effect of variation on total activity and any effect itself was very small [*F*_1,244_ = 0.448, *P* = 0.504; Eta2 (partial) 1.83 × 10^−3^, 95% CI [0.00, 1.00]]. The type of net used had a statistically significant main effect on total activity, and the effect size was large [*F*_2,244_ = 191.394, *P* < 0.001; Eta2 (partial) = 0.61, 95% CI [0.55, 1.00]]. A statistically significant interaction effect between strain and net distribution type on total activity was noted and the effect size was small [*F*_1,244_ = 6.999, *P* < 0.01; Eta2 (partial) = 0.05, 95% CI [0.01, 1.00]]. Estimated marginal mean values and their plausible ranges from the model output were generated for all levels of the two factors strain and net type and are shown in table S3 with confidence intervals at 95%. Post hoc pairwise comparisons between different levels of each factor were performed using “emmeans,” and confidence levels were adjusted because of variable group size using the Tukey method at 95% and are shown in table S4. For Kisumu, the difference in activity was not significant (*P* = 0.1386), presumably because this susceptible strain was affected by the amount of insecticide on both PFAS (+) and PFAS (−) nets. For the resistant Tiassalé strain, the difference in activity between PFAS (+) and PFAS (−) nets was found to be significant (*P* = 0.0001).

#### Spatiotemporal regional analysis of mean activity

**Fig. 8.**
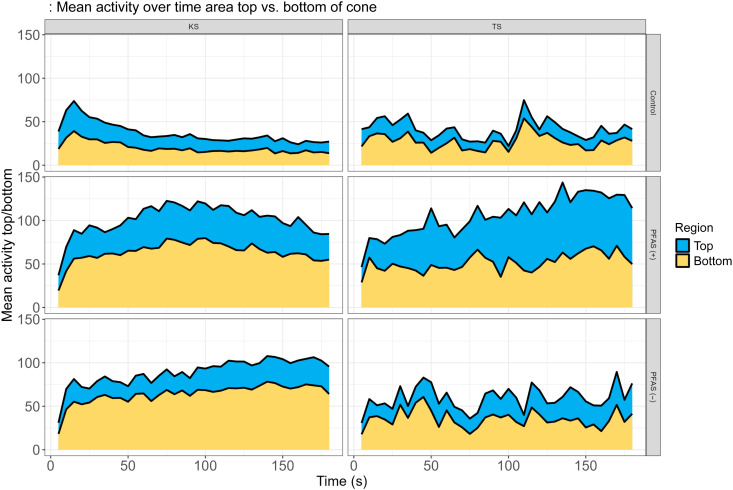
Mean activity over time in the top versus bottom of the cone by net type and mosquito strains. The stacked area plots show the mean mosquito activity over time (in seconds) in the top (blue) and bottom (yellow) regions of the cone for two mosquito strains: Kisumu (KS; left column) and Tiassalé (TS; right column). The activity was recorded during exposure over 3 min to three net types: control (top row), PFAS (+) net (middle row), and PFAS (−) net (bottom row). The *y* axis represents the mean activity in each region of the cone, highlighting differences in spatiotemporal distribution and overall activity levels for each strain and net type.

Analysis of mean activity over time in the top versus bottom regions of the cone highlighted contrasting behavior between Kisumu and Tiassalé mosquito strains during the 3-min exposure to control net, PFAS (+) net, and PFAS (−) net (Fig. 8). Kisumu strain, on a control net, had an initial rise in activity (within 25 s), declining over time to a low level, reflecting initial exploratory behavior and lack of stimuli from the net surface. However, exposure of Kisumu to PFAS (+) net resulted in a pronounced rise in activity which declined during the second half of the 3-min exposure (potentially due to the toxic insecticide exerting its effects), demonstrating strong excito-repellent effects of deltamethrin-treated nets. The PFAS (−) net elicited a similar response but with lower activity levels (particularly in the upper region of the cone, away from the net surface) and a slower increase in activity when compared with PFAS (+) net, potentially due to reduced irritancy due to the reduced deltamethrin bioavailability.

The Tiassalé strain on control net had a higher level of activity than the Kisumu strain, reflecting innate phenotypic differences in behavior between the strains. Exposure of Tiassalé to PFAS (+) net caused a gradual increase in activity over time. This increase was slower to accumulate compared with that of Kisumu, and the decline in activity during the second half of the experiment seen in Kisumu was not observed, indicating suppression of excito-repellent behavior due to resistance. With PFAS (−) net exposure on Tiassalé, activity was much lower than the PFAS (+) net, and no overall trend was observed. Activity in the top region was less pronounced, suggesting reduced bioavailability of the AI, which likely diminishes the irritancy effects of the newer formulation. These findings align with those of Jones *et al.* (*19*), showing stronger excito-repellent effects in susceptible mosquito strains and reduced responses in resistant populations.

Studies have shown that the irritancy and behavioral escape responses of mosquitoes, such as those to deltamethrin-treated papers, substantially influence their effectiveness, with resistant populations exhibiting diminished responses to insecticide exposure (*37*, *38*). Moreover, repeated exposure to pyrethroids like deltamethrin can lead to changes in mosquito behavior that reduce the efficacy of control tools (*39*). These findings emphasize the critical need to integrate strain-specific behavioral insights into ITNs design to ensure optimal effectiveness across diverse mosquito populations.

#### Behavioral state events over time

**Fig. 9.**
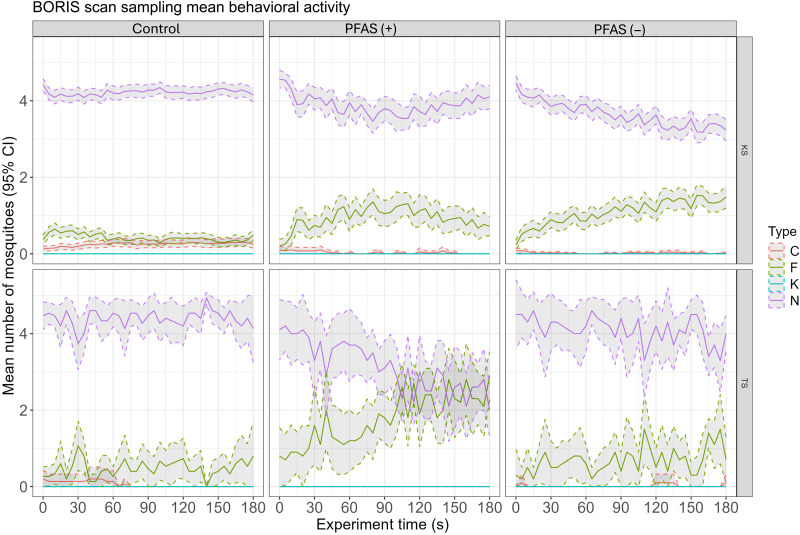
Mean behavioral activity of mosquitoes over 3 min during cone assay experiments, measured using BORIS scan sampling. The panels display the results for two mosquito strains Kisumu (KS) and Tiassalé (TS) under three treatments: negative control, PFAS (+) net, and PFAS (−) net. Behavioral states are represented as contacting the net (N; purple), flying (F; green), knocked down (K; blue), and resting on the cone (C; red). Solid lines indicate the mean number of mosquitoes in each behavioral category at each time point, with shaded regions representing the 95% confidence intervals (CI).

Scan-sampling analysis was performed to quantify mosquito behavioral states during cone assay video recordings, focusing on mosquito responses to ITNs and evaluating strain-specific differences in efficacy. Behavioral states of mosquitoes, categorized as contacting the net (N), flying (F), resting on the cone (C), or knocked down (K), were recorded using manual scan sampling at 5-s intervals during cone assay video recordings. Distinct patterns emerged between Kisumu and Tiassalé mosquito strains across treatments: negative control, PFAS (+) net, and PFAS (−) net (Fig. 9). For the negative control, activity was stable, with resting behaviors (C) predominating. It is notable that Tiassalé mosquitoes demonstrated more flight activity than Kisumu under control conditions, again suggesting differences in their innate behavioral response. In Kisumu mosquitoes, exposure to the PFAS (+) net led to a marked increase in flight activity (F), peaking at around 60 to 90 s before declining. This corresponds to the peak of activity observed in the automated image analysis in Fig. 8 and highlights the excito-repellent effects of deltamethrin, consistent with previous studies on ITN-induced behavioral responses (*16*–*20*, *37*, *39*). The PFAS (−) net elicited a similar, although slightly reduced, behavioral response in Kisumu mosquitoes, with a greater proportion retaining flight activity toward the end of the experiment, suggesting reduced excito-repellency (irritancy) compared with PFAS (+) net. In Tiassalé mosquitoes, exposure to PFAS (+) net resulted in earlier increases in flight activity, compared with that observed when exposed to PFAS (−) net. A corresponding steep fall in the number of mosquitoes at the net surface was also seen in PFAS (+) net. However, for PFAS (−) net, the number of mosquitoes at the net surface from the start of the experiment and onward declined only slightly. This indicates that the net surface of PFAS (−) net does not provoke as strong a behavioral response in Tiassalé as the PFAS (+) net. These findings also align with prior observations that excito-repellent effects are more pronounced in susceptible populations, while resistant strains exhibit attenuated responses (*20*).

## DISCUSSION

This study establishes a multidisciplinary platform for evaluating ITNs that combines chemical analysis of the active from macroscale content to microscale surface presentation, with video cone assays to evaluate mosquito behavior and mortality and with high-resolution, high-frame-rate imaging to study mosquito-ITN interactions. This multifaceted approach has revealed that the removal of PFAS-based coatings, mandated by growing environmental regulatory pressures (*29*), has introduced significant and previously underappreciated limitations to ITN performance. Our comparative analysis of PFAS-containing and PFAS-free PermaNet 2.0 formulations shows that insecticide presentation chemistry, not merely AI quantity, critically determines operational efficacy. Although both net types met World Health Organization (WHO) deltamethrin content specifications (*13*), PFAS-coated nets consistently demonstrated superior bio-efficacy against both susceptible and resistant *Anopheles* strains. These differences were most pronounced in the pyrethroid-resistant Busia, Tiassalé, and Tiefora strains, where PFAS-free nets exhibited distinct reduced 1-hour mortality, despite comparable levels of AI. These efficacy patterns were consistent with the resistance genotypes prevalent in these populations, notably the high frequencies of *Vgsc*-alleles, which are known to diminish pyrethroid binding affinity (*21*, *25*–*28*).

Chemical analysis confirmed that both net types contained the expected deltamethrin bioactive isomer without signs of degradation. However, PFAS-free nets showed greater variability in AI distribution and notably larger, more heterogeneous surface particulates. SEM and EDX analysis revealed not only differences in particulate morphology but also the absence of fluorine in PFAS (−) nets, marking the formulation shift (*9*). Raman microscopy further showed that PFAS (+) nets lacked crystalline signatures, suggesting that the deltamethrin existed in an amorphous or molecularly dispersed form, associated with higher bioavailability and toxicity (*30*–*36*). In contrast, PFAS (−) net contained mixed particulate populations, including crystalline form I, which is less bioactive. These structural differences translated into pronounced changes in mosquito behavior. Cone video analysis and scan sampling revealed that PFAS (+) nets triggered substantially more activity and irritancy, especially in the susceptible Kisumu strain compared with PFAS (−) nets. In the pyrethroid-resistant Tiassalé strain, PFAS (−) nets induced markedly lower movement, with fewer escape behaviors and prolonged net contact. These patterns were supported by statistically significant differences in total movement, spatial activity patterns, and behavioral state transitions (e.g., resting versus flight) (*19*, *37*–*39*).

These findings highlight the contribution of excito-repellency and sublethal incapacitation to ITN efficacy (*40*). While WHO Prequalification evaluation incorporates multiple bioassays, including cone tests, current frameworks primarily assess mortality and KD outcomes rather than explicitly quantifying behavioral endpoints. Our results suggest that behavioral responses may provide additional mechanistic insight into performance differences between nets, particularly under varying resistance backgrounds. We also note that the ViCTA approach is most applicable to irritant or rapidly acting compounds such as pyrethroids and may be less suitable for evaluating slow-acting toxicants.

Although standard WHO cone assays remain a valuable and sensitive tool for detecting changes in ITN efficacy, particularly through KD and mortality endpoints, the absence of a formalized framework for longitudinal reporting and standardized data integration may limit the early detection of performance shifts across product batches and time. Recent WHO postmarket surveillance (PMS) guidance encourages the integration of surface-level insecticide quantification, advanced imaging, wash-resistance testing, and behavioral telemetry; however, these elements have yet to be formally integrated into the PQT/VCP assessment framework (*41*). As a result, the performance of ITNs may decline undetected, particularly as formulation components are phased out because of environmental regulations, without proven, high-performing substitutes. Compounding the risk, global regulatory initiatives targeting PFAS, now exceeding seven million distinct compounds (*10*, *42*), may constrain the market’s capacity to develop sustainable, high-performance alternatives.

A practical next step could involve the development of standardized reporting pipelines that combine conventional bioassays with complementary behavioral and chemical metrics to enable cross-site comparison and long-term tracking of ITN performance. Implementation costs are modest where existing cone-test infrastructure and basic imaging setups are available, suggesting feasibility for integration into broader surveillance programs. Building on the validated outcomes of our study, we propose this framework as a forward-looking and practical solution to bridge key regulatory and operational gaps in vector control.

Our findings demonstrate the feasibility and value of a fully integrated, surface presentation-centric approach that aligns directly with the WHO’s evolving PMS agenda. This framework integrates surface bioavailability profiling with high-resolution imaging to reveal spatial heterogeneity in insecticide presentation that is not captured by conventional bulk chemical assays. Resolving these spatial inconsistencies provides mechanistic insight into why nominally equivalent nets can produce divergent behavioral and efficacy outcomes, particularly under resistant backgrounds where localized bioavailability may drive sublethal exposure. Coupling surface-resolved analysis with behavioral phenotyping provides complementary mechanistic insight alongside standard mortality endpoints, enabling quantitative assessment of KD, excito-repellency, and functional impairment across laboratory and semifield contexts. In parallel, life cycle–based surveillance encompassing net integrity, AI decay, and behavioral efficacy offers the longitudinal perspective needed to predict and monitor ITN performance under real-world conditions. The framework is designed to serve both research and development and regulatory functions, bridging prequalification and PMS within a unified, adaptable platform. Although now optimized for coated pyrethroid-based ITNs, the framework is conceptually adaptable to emerging technologies through modification of behavioral and chemical endpoints. Together, this integrated strategy provides a scalable platform linking mechanistic understanding with regulatory evaluation, with potential to strengthen evidence generation and refine WHO-aligned evaluation and surveillance across the product life cycle.

## MATERIALS AND METHODS

### Insecticidal netting

The procurement of the ITNs for this study is detailed by Vinit *et al.* (*6*). Unused ITNs manufactured in 2018 and 2019 were provided by Rotarians against Malaria PNG from consignments designated for various PNG provinces, whereas unused ITNs produced between 2007 and 2017 were sourced from villages or provincial health authorities across different region of PNG. All ITNs were stored in their original and unopened packaging (*6*). Four nets were randomly selected from each population of sampled ITNs with manufacturing date 2012 [PFAS (+) net] and manufacturing date 2019 [PFAS (−) net]. Nets remained sealed and stored under refrigeration. They are representative of these populations, as described in previous publications (*9*, *43*). Five swatches were taken from each panel of the nets and half of the net swatches were sent to Liverpool School of Tropical Medicine (LSTM) for analysis, with the other half used for different purposes.

### Mosquito strains

Four mosquito strains were used in this study: the susceptible Kisumu strain alongside the resistant Tiassalé, Tiefora, and Busia strains. The Tiassalé strain was colonized from a population of *An. gambiae* sensu stricto (ss) in Côte d’Ivoire (*23*). It is resistant to the four insecticide classes used for malaria control and is maintained at LSTM with 6-monthly selection using 0.05% deltamethrin (*23*). The Tiefora strain was colonized from *Anopheles coluzzii* mosquitoes caught in the Banfora district of Burkina Faso in 2018 (*24*). It also exhibits resistance to four classes of insecticide and is maintained with 6-monthly selection using 0.05% deltamethrin for 4 hours (*44*). The Busia strain was established from field collections of *An. gambiae* ss in Busia, Uganda, in 2018 (*21*). It is maintained at LSTM and is selected with 0.05% deltamethrin every five generations (*20*). All mosquitoes were reared in environmentally controlled insectaries with temperatures between 25° and 27°C and relative humidity of 70 to 80%. Unfed female mosquitoes between 3 and 5 days old were used in tests.

### WHO cone

Eight net swatches from 2012 and 11 from 2019 were evaluated using the WHO cone assay (*12*). Net swatches were secured to a plastic frame with 11- to 12-cm-diameter holes, and the cones were placed over the holes. The frame was positioned at a 45° angle, and five female mosquitoes were placed in the cone for 3 min of exposure to the net. The mosquitoes were transferred to cups with access to sugar water. KD and mortality were recorded at 60 min of the assay and following a 24-hour recovery period, respectively. Each swatch was assayed five times with a total of 25 mosquitoes. Negative controls were run at the same time using untreated net swatches. The assays were carried out in a controlled environment with temperatures of 27° ± 2°C and relative humidity of 80 ± 20%.

### Chemical analyses

#### Reagents

Technical-grade deltamethrin (98.6%; PESTANAL, Sigma-Aldrich, product no. 45423) was used as the analytical standard and dicyclohexyl phthalate (DCP; 99%; Sigma-Aldrich, product no. 306150) as the internal standard. Liquid chromatography (LC)–MS–grade acetonitrile (≥99.8%; Fisher Scientific, product code 15402867), LC-MS–grade water (Fisher Chemical, product code 10777404), and heptane (>99%; Thermo Scientific Acros, product code 10510781) were obtained from Fisher Scientific, and LC-MS–grade 1-propanol (>99%; Thermo Scientific, product code 10791464) was purchased from Thermo Scientific.

#### Sample preparation

The quantification of total deltamethrin content was performed using an established extraction protocol (*45*). Three independent net samples per manufacturing batch (distinct net IDs) were analyzed. For each net sample (~50 cm^2^; ~0.1500 g), an extraction solution containing DCP (100 μg/ml) in a 1:9 (v/v) mixture of 1-propanol and heptane was prepared. Five milliliters of extraction solution were added to each sample in a 10-ml glass tube, and samples were heated at 85°C for 45 min. Extractions were conducted over four sequential rounds using fresh extraction solution and clean glassware each time; between rounds, net pieces were rinsed with cold heptane to remove residual solvent.

#### Sample processing

After each extraction, 1 ml of the resulting solution was transferred to a new 10-ml glass tube and evaporated under compressed air at 60°C. The evaporated residue was resuspended in 1 ml of acetonitrile, vortexed for 1 min at 2,500 rpm, and transferred to a 1.5-ml centrifuge tube. The samples were centrifuged at 13,000 rpm for 20 min, and 100 μl of the supernatant was pipetted into HPLC vials for analysis.

#### HPLC analysis

HPLC was performed using a Dionex UltiMate 3000 system equipped with a Hypersil GOLD C18 column (75 Å, 250 mm by 4.6 mm, 5-μm particle size). The mobile phase consisted of a 70:30 (v/v) acetonitrile:water mixture with a flow rate of 1 ml/min. The injection volume was 20 μl, and the total run time was 25 min. Detection was carried out at a wavelength of 232 nm. Peak areas were integrated using Chromeleon 7.2.9 software. Deltamethrin content (grams per kilogram) was calculated using the equation

$$I=\left(\frac{x}{a}\right)\times \left(\frac{0.001}{m}\right)\times C\times f$$

where *I* is the insecticide content in grams per kilogram, *x* is the sample peak area for deltamethrin, *a* is the slope of the deltamethrin standard curve, *m* is the mass of the net sample, *C* is the internal standard correction factor, and *f* is the sample dilution factor. The deltamethrin content extracted in each round was combined to determine the total deltamethrin content in the net sample. Data in table S1 are reported in both grams per kilogram and milligrams per square meter. Reporting in grams per kilogram is considered more precise, as weighing each individual net provides greater accuracy compared to assuming that the cutting method consistently produces perfectly sized samples. Additionally, the results in grams per kilogram better align with the intended target dose. Across all cases, more than 97% of the AI; deltamethrin was extracted during the first extraction round.

#### GC-MS analysis

GC-MS analysis was conducted using an Agilent GC-MS system fitted with an HP-5MS UI capillary column (30 m length, 0.25 mm inner diameter, and 0.25 μm film thickness). Helium served as the carrier gas at a constant flow rate of 1.2 ml/min. Samples (1 μl) were injected using a split injection mode (50:1 split ratio), with an inlet temperature set at 280°C. The split flow was maintained at 60 ml/min, septum purge flow was maintained at 3 ml/min, and a gas saver flow of 15 ml/min was applied after 2 min. The oven temperature program began at 50°C (held for 1 min), then increased at a rate of 20°C/min to 300°C, and was held at this final temperature for 10 min. This resulted in a total run time of 23.5 min. Detection involved a flame ionization detector operating at 300°C with gas flows set as follows: air at 400 ml/min, hydrogen at 30 ml/min, and nitrogen makeup gas at 25 ml/min. MS detection used electron ionization at 70 eV. Data acquisition started after a solvent delay of 3 min, using single ion monitoring mode at mass/charge ratio 272.00 with a dwell time of 100 ms, scanning a mass range from 17 to 500 u at a scan speed of 1562 μ/s. Instrument control, data acquisition, and processing were performed using Agilent’s proprietary software. This analytical method allowed for qualitative identification of the target compounds.

#### Raman spectroscopy

All Raman spectra were measured with a Renishaw inVia confocal micro-spectrometer with a 785-nm laser with 26.4 mW at source. A 50× objective was used for the measurements on single particulates on the ITNs. The two forms of neat deltamethrin, unheated (form I) and melted (form II) deltamethrin, were placed on a gold substrate for Raman measurement. All data were analyzed using MATLAB R2023a. Data processing was done as follows: Smoothing with MATLAB built-in Savitzky-Golay smoothing function (window size of 5, first order), baseline correction using a polynomial baseline correction with asymmetric least squares algorithm as in the cluster-toolbox-v2.0 (https://github.com/Biospec/cluster-toolbox-v2.0) and normalization using the vectornorm function in the CLIRSPEC-Summer-School-2015 (v1.0) package (*46*).

#### SEM and EDX spectroscopy

All SEM images and EDX spectra were acquired using a JEOL JSM 7001F SEM System with using the Oxford Instruments INCA software for SEM images acquisitions. All EDX data were acquired and analyzed with the Oxford Instruments AZtec software.

#### Polarized light microscopy

An AmScope ME580T PZ 2 L microscope coupled with crossed polarizers and a digital camera was used for analyzing the phase transition of the deltamethrin form I upon heating and recooling. The thin film was prepared according to method described by Yang *et al.* (*31*) using 98.6% deltamethrin purchased from Sigma-Aldrich. Example images were taken with a 10× objective.

### VCTs and analysis

VCT experiments were conducted on a proportion of cone assays using the simple augmentation of the WHO cone assay described by Hughes *et al.* (*18*) and Jones *et al.* (*19*), initially using the susceptible Kisumu strain (*18*, *19*). High mortality rates observed in this strain prompted further experiments using the resistant Tiassalé strain to try to more clearly delineate responses between the different net compositions. The VCT simply adds video recording of the 3-min cone assay by a smartphone attached to the cone board along with optional presentation of a host stimulus. No host stimulus signal was provided in these VCT experiments to allow comparison to the results of standard cone assays performed on the other strains. These recordings were later analyzed using two methods. The first method, scan sampling, is a manual assessment of mosquito state during the cone assay video recordings and was performed by pausing the video recording at 5-s intervals and recording the current behavioral state of the mosquitoes at each time point (*18*). The behaviors were recorded using the Behavior Observation Research Interactive Software (BORIS) ethology recording software (*47*) and is encoded as the number of mosquitoes in the following unique behavioral states: contacting the net (N), flying within the cone (F), resting on the cone surface (C), and knocked down (K). Scan-sampling analysis of each video recording resulted in 37 time points of behavioral states that were exported to Microsoft Excel files, before further analysis using custom R scripts.

The second analysis method is an automated process using the ViCTA software developed at LSTM. The software detects mosquito movement within the cone using a machine-vision approach using background subtraction segmentation by a Mixture of Gaussian model (*19*). Video frames from recorded cone assays are analyzed at 0.1-s intervals during the 3-min experiment, and a count of the number of moving mosquitoes and their location is stored. The 1800 analyzed frames are aggregated into 5-s interval counts to compare with the scan-sampling results. Total mosquito activity (the number of movements detected during the assay) is recorded along with the positions of moving mosquitoes in the cone (assigned to upper or lower half regions of the cone). The output of a single recording is pasted into a custom Microsoft Excel data entry template for later analysis by custom R scripts after completion of all experiments. The two assessment methods (BORIS and ViCTA) measure slightly different parameters (the amount and location of movement versus the behavioral state of the mosquitoes) during the cone assay and complement each other, yielding insights into the mosquito response to the ITN during the cone assays.

### Data and statistical analysis

Data analysis was implemented using R (version 4.2.2, R Core Team, 2022) (*48*). Charts were created using the “ggplot2” package (version 3.5.0) (*49*). Descriptive statistics were generated using number of observations, mean, SD, and 95% confidence intervals. Data were assessed for homogeneity of variance and normality across treatment groups using Levene’s test for homogeneity due to unequal sample sizes. Mean distributions of total activity were compared using Welch’s independent *t* tests. Cohen’s *D* measure was used to assess the effect size differences and were labeled following Field’s recommendations (*50*). A two-way ANOVA was used to analyze the main and interaction effects of strain and net distribution type on the total mosquito movement. Residuals of the ANOVA model were manually analyzed with a QQ normal plot and assessed using the Shapiro-Wilk test. Estimated marginal means were calculated using emmeans package (*51*) on the ANOVA model output to assess the relative contribution of levels in the strain and net distribution type groups and their interactions. Post hoc pairwise comparisons between different levels of each factor were performed using the emmeans package (*51*), and confidence levels adjusted were because of variable group size using the Tukey method at 95%.

## References

[1] World Health Organization (WHO), “World malaria report 2024: Addressing inequity in the global malaria response” (WHO, 2024).

[2] S. Bhatt, D. J. Weiss, E. Cameron, D. Bisanzio, B. Mappin, U. Dalrymple, K. Battle, C. L. Moyes, A. Henry, P. A. Eckhoff, E. A. Wenger, O. Briet, M. A. Penny, T. A. Smith, A. Bennett, J. Yukich, T. P. Eisele, J. T. Griffin, C. A. Fergus, M. Lynch, F. Lindgren, J. M. Cohen, C. L. J. Murray, D. L. Smith, S. I. Hay, R. E. Cibulskis, P. W. Gething, The effect of malaria control on *Plasmodium falciparum* in Africa between 2000 and 2015. Nature 526, 207–211 (2015). 26375008 10.1038/nature15535 PMC4820050

[3] S. W. Lindsay, M. B. Thomas, I. Kleinschmidt, Threats to the effectiveness of insecticide-treated bednets for malaria control: Thinking beyond insecticide resistance. Lancet Glob. Health 9, e1325–e1331 (2021). 34216565 10.1016/S2214-109X(21)00216-3

[4] H. Ranson, N. Lissenden, Insecticide resistance in African *Anopheles* mosquitoes: A worsening situation that needs urgent action to maintain malaria control. Trends Parasitol. 32, 187–196 (2016). 26826784 10.1016/j.pt.2015.11.010

[5] M. W. Hetzel, J. Pulford, Y. Ura, S. Jamea-Maiasa, A. Tandrapah, N. Tarongka, L. Lorry, L. J. Robinson, K. Lilley, L. Makita, P. M. Siba, I. Mueller, Insecticide-treated nets and malaria prevalence, Papua New Guinea, 2008-2014. Bull. World Health Organ. 95, 695–705B (2017). 29147042 10.2471/BLT.16.189902 PMC5689189

[6] R. Vinit, L. Timinao, N. Bubun, M. Katusele, L. J. Robinson, P. Kaman, M. Sakur, L. Makita, L. Reimer, L. Schofield, W. Pomat, I. Mueller, M. Laman, T. Freeman, S. Karl, Decreased bioefficacy of long-lasting insecticidal nets and the resurgence of malaria in Papua New Guinea. Nat. Commun. 11, 3646 (2020). 32686679 10.1038/s41467-020-17456-2 PMC7371689

[7] M. W. Hetzel, H. Morris, N. Tarongka, C. Barnadas, J. Pulford, L. Makita, P. M. Siba, I. Mueller, Prevalence of malaria across Papua New Guinea after initial roll-out of insecticide-treated mosquito nets. Trop. Med. Int. Health 20, 1745–1755 (2015). 26427024 10.1111/tmi.12616

[8] M. Katusele, S. Lagur, N. Endersby-Harshman, S. Demok, J. Goi, N. Vincent, M. Sakur, A. Dau, L. Kilepak, S. Gideon, C. Pombreaw, L. Makita, A. Hoffmann, L. J. Robinson, M. Laman, S. Karl, Insecticide resistance in malaria and arbovirus vectors in Papua New Guinea, 2017-2022. Parasit. Vectors 15, 426 (2022). 36376932 10.1186/s13071-022-05493-3 PMC9664807

[9] N. Bubun, E. Anetul, M. Koinari, T. W. Freeman, S. Karl, Coating formulation change leads to inferior performance of long-lasting insecticidal nets in Papua New Guinea. Malar. J. 21, 349 (2022). 36424604 10.1186/s12936-022-04392-3 PMC9685832

[10] Z. Wang, A. M. Buser, I. T. Cousins, S. Demattio, W. Drost, O. Johansson, K. Ohno, G. Patlewicz, A. M. Richard, G. W. Walker, G. S. White, E. Leinala, A New OECD Definition for Per- and Polyfluoroalkyl Substances. Environ. Sci. Technol. 55, 15575–15578 (2021). 34751569 10.1021/acs.est.1c06896

[11] E. L. Schymanski, J. Zhang, P. A. Thiessen, P. Chirsir, T. Kondic, E. E. Bolton, Per- and polyfluoroalkyl Substances (PFAS) in PubChem: 7 Million and Growing. Environ. Sci. Technol. 57, 16918–16928 (2023). 37871188 10.1021/acs.est.3c04855 PMC10634333

[12] World Health Organization (WHO), “WHO specifications and evaluations for public health pesticides: Deltamethrin-coated long-lasting insecticidal nets (LN)” (WHO, 2024).

[13] O. Skovmand, D. M. Dang, T. Q. Tran, R. Bossellman, S. J. Moore, From the factory to the field: Considerations of product characteristics for insecticide-treated net (ITN) bioefficacy testing. Malar. J. 20, 363 (2021). 34488778 10.1186/s12936-021-03897-7 PMC8422710

[14] C. L. Moyes, R. S. Lees, C. Yunta, K. J. Walker, K. Hemmings, F. Oladepo, P. A. Hancock, D. Weetman, M. J. I. Paine, H. M. Ismail, Assessing cross-resistance within the pyrethroids in terms of their interactions with key cytochrome P450 enzymes and resistance in vector populations. Parasit. Vectors 14, 115 (2021). 33602297 10.1186/s13071-021-04609-5 PMC7893915

[15] V. A. Ingham, A. Anthousi, V. Douris, N. J. Harding, G. Lycett, M. Morris, J. Vontas, H. Ranson, A sensory appendage protein protects malaria vectors from pyrethroids. Nature 577, 376–380 (2020). 31875852 10.1038/s41586-019-1864-1 PMC6974402

[16] E. Sherrard-Smith, J. E. Skarp, A. D. Beale, C. Fornadel, L. C. Norris, S. J. Moore, S. Mihreteab, J. D. Charlwood, S. Bhatt, P. Winskill, J. T. Griffin, T. S. Churcher, Mosquito feeding behavior and how it influences residual malaria transmission across Africa. Proc. Natl. Acad. Sci. U.S.A. 116, 15086–15095 (2019). 31285346 10.1073/pnas.1820646116 PMC6660788

[17] E. K. Thomsen, G. Koimbu, J. Pulford, S. Jamea-Maiasa, Y. Ura, J. B. Keven, P. M. Siba, I. Mueller, M. W. Hetzel, L. J. Reimer, Mosquito behavior change after distribution of bednets results in decreased protection against malaria exposure. J Infect Dis 215, 790–797 (2017). 28007921 10.1093/infdis/jiw615 PMC5388271

[18] A. Hughes, A. Matope, M. Emery, K. Steen, G. Murray, H. Ranson, P. J. McCall, G. M. Foster, A closer look at the WHO cone bioassay: Video analysis of the hidden effects of a human host on mosquito behaviour and insecticide contact. Malar. J. 21, 208 (2022). 35778744 10.1186/s12936-022-04232-4 PMC9248144

[19] J. Jones, A. Matope, P. Barreaux, K. Gleave, K. Steen, H. Ranson, P. J. McCall, G. M. Foster, Video augmentation of the WHO cone assay to quantify mosquito behavioural responses to insecticide-treated nets. Parasit. Vectors 16, 420 (2023). 37968752 10.1186/s13071-023-06029-z PMC10652617

[20] E. Reid, F. Mechan, J. Jones, A. Lynd, J. Hemingway, P. McCall, D. Weetman, Behavioural responses of *Anopheles gambiae* to standard pyrethroid and PBO-treated bednets of different operational ages. Curr. Res. Parasitol. Vector Borne Dis. 6, 100227 (2024). 39582751 10.1016/j.crpvbd.2024.100227 PMC11584737

[21] A. Oruni, A. Lynd, H. Njoroge, I. Onyige, A. E. Van’t Hof, E. Matovu, M. J. Donnelly, Pyrethroid resistance and gene expression profile of a new resistant *An. gambiae* colony from Uganda reveals multiple resistance mechanisms and overexpression of Glutathione-S-Transferases linked to survival of PBO-pyrethroid combination. Wellcome Open Res 9, 13 (2024). 38813466 10.12688/wellcomeopenres.19404.2 PMC11134160

[22] A. V. C. Edi, B. P. N’Dri, M. Chouaibou, F. B. Kouadio, P. Pignatelli, G. Raso, D. Weetman, B. Bonfoh, First detection of N1575Y mutation in pyrethroid resistant *Anopheles gambiae* in Southern Côte d’Ivoire. Wellcome Open Res 2, 71 (2017). 29018842 10.12688/wellcomeopenres.12246.1 PMC5627500

[23] C. V. Edi, B. G. Koudou, C. M. Jones, D. Weetman, H. Ranson, Multiple-insecticide resistance in *Anopheles gambiae* mosquitoes Southern Côte d’Ivoire. Emerg. Infect. Dis. 18, 1508–1511 (2012). 22932478 10.3201/eid1809.120262 PMC3437712

[24] J. Williams, V. A. Ingham, M. Morris, K. H. Toe, A. S. Hien, J. C. Morgan, R. K. Dabire, W. M. Guelbeogo, N. Sagnon, H. Ranson, Sympatric populations of the *Anopheles gambiae* complex in southwest Burkina Faso evolve multiple diverse resistance mechanisms in response to intense selection pressure with pyrethroids. Insects 13, 247 (2022). 35323544 10.3390/insects13030247 PMC8955173

[25] M. J. Burton, I. R. Mellor, I. R. Duce, T. G. Davies, L. M. Field, M. S. Williamson, Differential resistance of insect sodium channels with kdr mutations to deltamethrin, permethrin and DDT. Insect Biochem. Mol. Biol. 41, 723–732 (2011). 21640822 10.1016/j.ibmb.2011.05.004

[26] C. S. Clarkson, A. Miles, N. J. Harding, A. O. O’Reilly, D. Weetman, D. Kwiatkowski, M. J. Donnelly, Anopheles gambiae 1000 Genomes Consortium, The genetic architecture of target-site resistance to pyrethroid insecticides in the African malaria vectors *Anopheles gambiae* and *Anopheles coluzzii*. Mol. Ecol. 30, 5303–5317 (2021). 33590926 10.1111/mec.15845 PMC9019111

[27] L. Grigoraki, R. Cowlishaw, T. Nolan, M. Donnelly, G. Lycett, H. Ranson, CRISPR/Cas9 modified *An. gambiae* carrying *kdr* mutation L1014F functionally validate its contribution in insecticide resistance and combined effect with metabolic enzymes. PLOS Genet. 17, e1009556 (2021). 34228718 10.1371/journal.pgen.1009556 PMC8284791

[28] N. K. Yellapu, J. Gopal, G. Kasinathan, J. Purushothaman, Molecular modelling studies of kdr mutations in voltage gated sodium channel revealed significant conformational variations contributing to insecticide resistance. J. Biomol. Struct. Dyn. 36, 2058–2069 (2018). 28608751 10.1080/07391102.2017.1341338

[29] Vestergaard, Response to Bloomberg article regarding malaria resurgence in Papua New Guinea and long-lasting insecticide-treated nets (LLINs). Company statement (23 February 2024).

[30] N. Bubun, T. W. Freeman, M. Laman, S. Karl, Effect of short-term heating on bioefficacy of deltamethrin-coated long-lasting insecticidal nets. Am. J. Trop. Med. Hyg. 106, 828–830 (2021). 34929669 10.4269/ajtmh.21-0613 PMC8922514

[31] J. Yang, B. Erriah, C. T. Hu, E. Reiter, X. Zhu, V. Lopez-Mejias, I. P. Carmona-Sepulveda, M. D. Ward, B. Kahr, A deltamethrin crystal polymorph for more effective malaria control. Proc. Natl. Acad. Sci. U.S.A. 117, 26633–26638 (2020). 33046642 10.1073/pnas.2013390117 PMC7604418

[32] T. Dong, L. Lin, Y. He, P. Nie, F. Qu, S. Xiao, Density functional theory analysis of deltamethrin and its determination in strawberry by surface enhanced raman spectroscopy. Molecules 23, 1458 (2018). 29914118 10.3390/molecules23061458 PMC6100570

[33] D. Tuschel, Why Are the Raman Spectra of Crystalline and Amorphous Solids Different? Spectroscopy 32, 26–33 (2017).

[34] P. M. A. Sherwood, *Vibrational Spectroscopy of Solids* (Syndics of the Cambridge University Press, 1972).

[35] H. Zhang, P. Nie, Z. Xia, X. Feng, X. Liu, Y. He, Rapid quantitative detection of deltamethrin in *Corydalis yanhusuo* by SERS coupled with multi-walled carbon nanotubes. Molecules 25, 4081 (2020). 32906783 10.3390/molecules25184081 PMC7570915

[36] B. Erriah, X. Zhu, C. T. Hu, B. E. Kahr, A. Shtukenberg, M. D. Ward, Crystallography of contemporary contact insecticides. Insects 13, 292 (2022). 35323590 10.3390/insects13030292 PMC8949367

[37] M. Kongmee, A. Prabaripai, P. Akratanakul, M. J. Bangs, T. Chareonviriyaphap, Behavioral responses of *Aedes aegypti* (Diptera: Culicidae) exposed to deltamethrin and possible implications for disease control. J. Med. Entomol. 41, 1055–1063 (2004). 15605644 10.1603/0022-2585-41.6.1055

[38] M. Mulatier, C. Pennetier, A. Porciani, F. Chandre, L. Dormont, A. Cohuet, Prior contact with permethrin decreases its irritancy at the following exposure among a pyrethroid-resistant malaria vector *Anopheles gambiae*. Sci. Rep. 9, 8177 (2019). 31160750 10.1038/s41598-019-44633-1 PMC6546682

[39] M. G. Machani, E. Ochomo, F. Amimo, W. R. Mukabana, A. K. Githeko, G. Yan, Y. A. Afrane, Behavioral responses of pyrethroid resistant and susceptible *Anopheles gambiae* mosquitoes to insecticide treated bed net. PLOS ONE 17, e0266420 (2022). 35390050 10.1371/journal.pone.0266420 PMC8989192

[40] World Health Organization (WHO), “WHO guideline for the prequalification assessment of insecticide-treated nets” (WHO, 2023).

[41] World Health Organization (WHO), “Considerations for post-market surveillance (PMS) of WHO prequalified insecticide treated nets (ITNs)” (WHO, 2025).

[42] Organisation for Economic Co-operation and Development (OECD), “Global database of per- and polyfluoroalkyl substances (PFAS)” (OECD, 2023).

[43] S. G. Mbwambo, N. Bubun, E. Mbuba, J. Moore, K. Mbina, D. Kamande, M. Laman, E. Mpolya, O. G. Odufuwa, T. Freeman, S. Karl, S. J. Moore, Comparison of cone bioassay estimates at two laboratories with different *Anopheles* mosquitoes for quality assurance of pyrethroid insecticide-treated nets. Malar. J. 21, 214 (2022). 35799172 10.1186/s12936-022-04217-3 PMC9264565

[44] J. Bagi, N. Grisales, R. Corkill, J. C. Morgan, S. N’Fale, W. G. Brogdon, H. Ranson, When a discriminating dose assay is not enough: Measuring the intensity of insecticide resistance in malaria vectors. Malar. J. 14, 210 (2015). 25985896 10.1186/s12936-015-0721-4 PMC4455279

[45] K. J. Walker, C. T. Williams, F. O. Oladepo, J. Lucas, D. Malone, M. J. I. Paine, H. M. Ismail, A high-throughput HPLC method for simultaneous quantification of pyrethroid and pyriproxyfen in long-lasting insecticide-treated nets. Sci. Rep. 12, 9715 (2022). 35690679 10.1038/s41598-022-13768-z PMC9188574

[46] A. Henderson, clirspec-summer-school: CLIRSPEC-Summer-School-2015, Zenodo (2016); 10.5281/zenodo.57398.

[47] O. Friard, M. Gamba, BORIS: A free, versatile open‐source event‐logging software for video/audio coding and live observations. Methods Ecol. Evol. 7, 1325–1330 (2016).

[48] R Core Team, R: A Language and Environment for Statistical Computing (R Foundation for Statistical Computing, 2022).

[49] H. Wickham, *ggplot2: Elegant Graphics for Data Analysis* (Springer-Verlag, 2016).

[50] A. Field, *Discovering Statistics Using IBM SPSS Statistics* (Sage Publications Limited, 2024).

[51] R. Lenth, emmeans: Estimated marginal means, aka least-squares means, R package version 1.8.5, CRAN (2023).

