# Supplementary material for: Multimodal platform for ITN efficacy: Surface chemistry, bioavailability, and mosquito behavior
Source: Sci Adv. 2026 Apr 8;12(15):eaeb2023. doi: 10.1126/sciadv.aeb2023 (PMC13060586; doi:10.1126/sciadv.aeb2023)
Supplement: Supplementary file 1 — Figs. S1 and S2 Tables S1 to S4 Legends for files S1 to S3 [file sciadv.aeb2023_sm.pdf]

Science Advances
AAAS

Supplementary Materials for

Multimodal platform for ITN efficacy: Surface chemistry, bioavailability, and mosquito behavior

Hanafy M. Ismail *et al.*

Corresponding author: Hanafy M. Ismail, hanafy.ismail@lstmed.ac.uk; Rasmita Raval, raval@liverpool.ac.uk

Sci. Adv. **12**, eaeb2023 (2026)
DOI: 10.1126/sciadv.aeb2023

The PDF file includes:

Figs. S1 and S2
Tables S1 to S4
Legends for files S1 to S3

Other Supplementary Material for this manuscript includes the following:

Files S1 to S3

Supplementary figures:

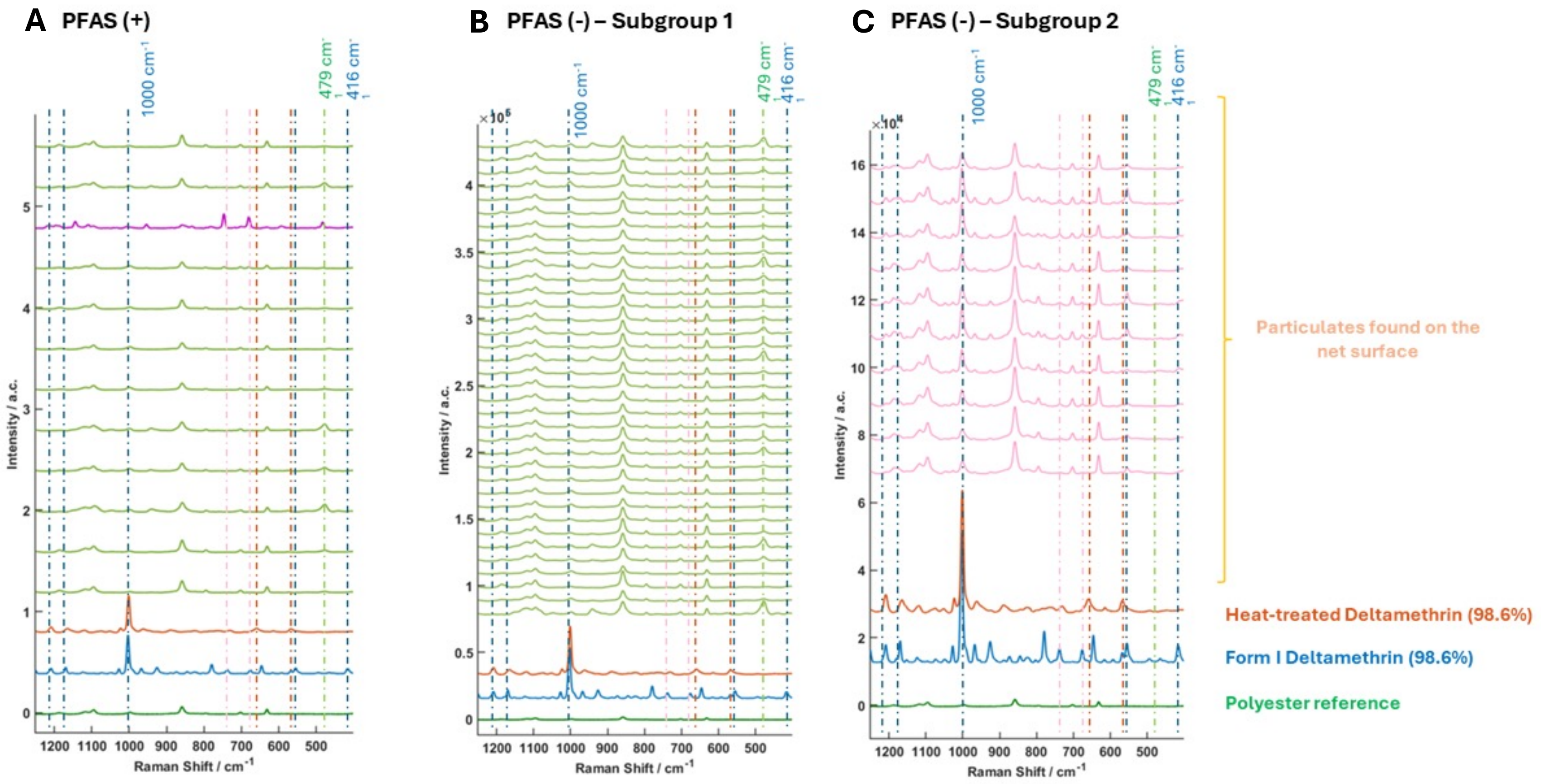

Fig. S1. Raman spectra of individual surface particulates from PFAS (+) and PFAS (-) nets. Stack of spectra of individual particulates from (a) PFAS (+) nets and (b) PFAS (-) nets, which subdivide into subgroup 1 and subgroup 2. For each group, representative spectra from Form I deltamethrin (blue trace, purity 98.6%), heat-treated deltamethrin (orange trace, purity 98.6%), and polyester reference (green trace) are shown for comparison. Prominent Raman shifts at 1000, 479, and 416 cm^-1 help distinguish between polymer, insecticide, and surface particulates.

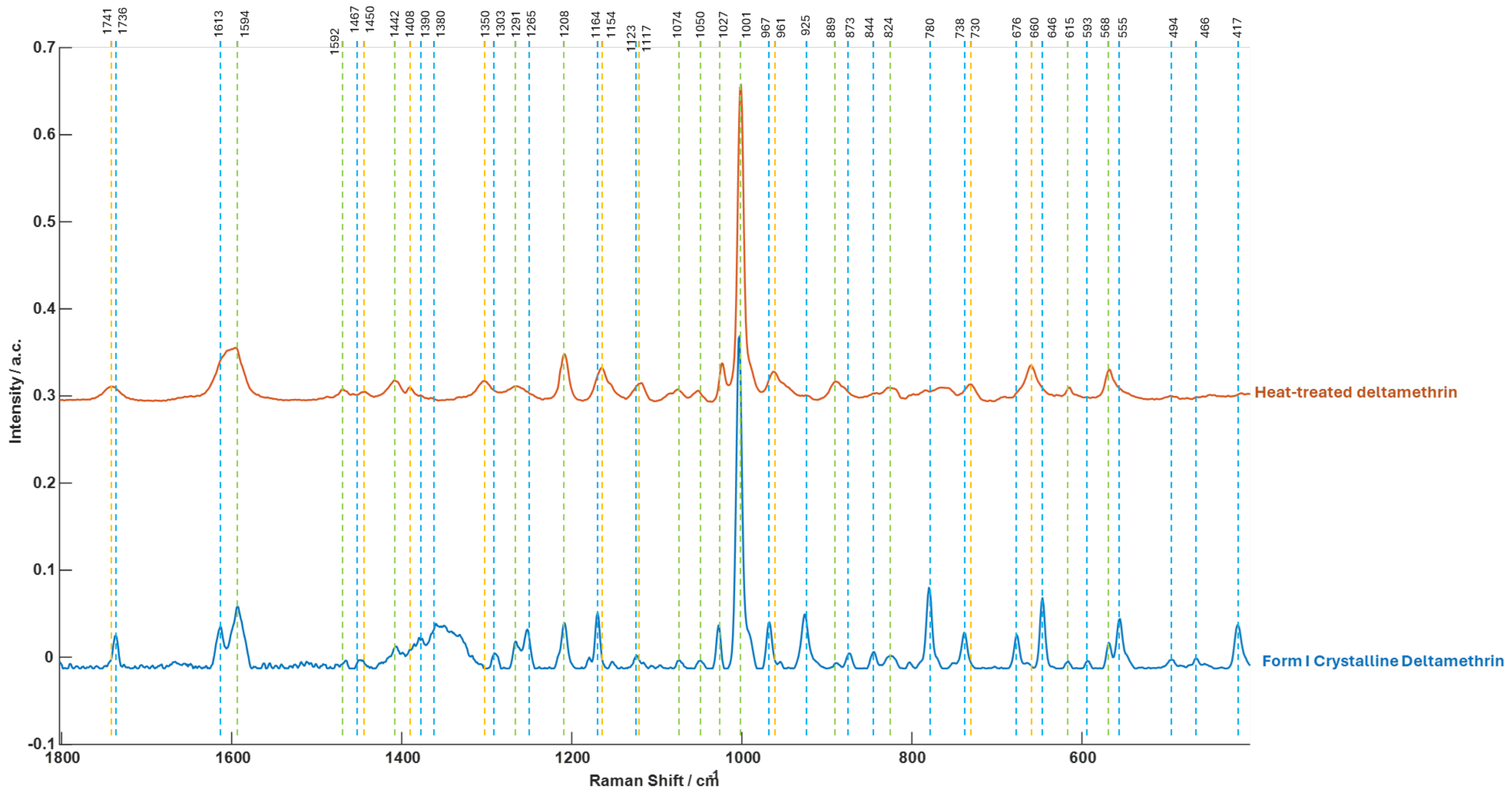

Fig. S2. Raman spectral comparison of heat-treated deltamethrin and Form I crystalline deltamethrin. Raman spectral comparison of heat-treated deltamethrin (orange trace) and Form I crystalline deltamethrin (blue trace). Characteristic peaks (indicated by dotted vertical lines) highlight spectral differences caused by heat treatment, reflecting changes in crystalline structure (polymorphs).

Supplementary tables:

Table S1.

Deltamethrin content analysis of polyester nets manufactured in 2012 (PFAS (+)) and 2019 (PFAS (-)). Data include sample IDs, denier, target and measured deltamethrin content (mg/m²), average dosage, standard deviation (Std. Dev), and percentage relative standard deviation (%RSD).

Sample type	Year of Manufacture	Sample ID	Denier	Target deltamethrin content [mg/m^2] ± 25%	Deltamethrin content [mg/m^2]	Average Dosage [mg/m^2]	Std. Dev	%RSD
PFAS (+)	2012	001 B	75	54 (40.5-67.5)	42.90	45.8 ± 2.5	2.54	5.54
		003 E	75	54 (40.5-67.5)	47.70			
		004 A	75	54 (40.5-67.5)	46.73			
PFAS (-)	2019	005 B	75	54 (40.5-67.5)	71.29	58.8 ± 11.1	11.07	18.81
		006 B	75	54 (40.5-67.5)	55.15			
		008 D	75	54 (40.5-67.5)	50.11			

Table S2.

Video cone test analysis (ViCTA) descriptive results. Mean total detected mosquito movement events using ViCTA method on treated nets manufactured in 2012 (PFAS (+)) and 2019 (PFAS (-)), and on untreated Control nets. KS = Kisumu susceptible strain, TS = Tiassalé resistant strain. N = number of replicates, SE = standard error, 95% confidence interval (lower and upper bounds, respectively).

**Strain**	**Net type**	**Mean total activity**	N	SE	**95% CI Lower**	**95% CI Upper**
KS	Control	1324.42	120	67.80	1190.18	1458.66
KS	PFAS+	3628.30	40	160.36	3303.94	3952.66
KS	PFAS-	3211.65	55	111.96	2987.19	3436.12
TS	Control	1484.60	15	228.27	995.02	1974.18
TS	PFAS+	3804.00	10	167.98	3424.00	4184.00
TS	PFAS-	2141.60	10	233.54	1613.29	2669.91

Table S3.

Estimated marginal means from a two-way ANOVA model (95% confidence). Estimated model-adjusted marginal means (EMM) of detected mosquito movement in the ViCTA method on treated nets manufactured in 2012 (PFAS (+)) and 2019 (PFAS (-)), and on untreated Control nets. KS = Kisumu susceptible strain, TS = Tiassalé resistant strain, SE = standard error, df = degrees of freedom, 95% confidence interval (lower and upper bounds, respectively).

Strain	Net type	**EMM**	**SE**	**df**	**lower.CL**	**upper.CL**
KS	Control	1324	74.2	244	1178	1471
TS	Control	1485	210	244	1071	1898
KS	PFAS+	3628	128.6	244	3375	3882
KS	PFAS-	3212	109.7	244	2996	3428
TS	PFAS+	3804	257.2	244	3297	4311
TS	PFAS-	2142	257.2	244	1635	2648

Table S4.
***Post-hoc* pairwise comparisons of model parameter estimates.** Mean differences between contrasts and estimated values at the upper and lower confidence bounds, respectively. Significant p-values are indicated in bold. Confidence intervals were adjusted using Tukey's method for multiple comparisons.

Contrast	**Mean difference**	**95% Confidence Interval**	Adjusted p-value
KS Control: TS Control	-160	(-799.9, 479.6)	0.9795
KS Control: KS PFAS+	-2304	(-2730.4, -1877.4)	**<.0001**
KS Control: KS PFAS-	-1887	(-2267.6, -1506.9)	**<.0001**
KS PFAS+: KS PFAS-	417	(-68.8, 902.1)	0.1386
TS Control: TS PFAS+	-2319	(-3273.1, -1365.7)	**<.0001**
TS Control: TS PFAS-	-657	(-1610.7, 296.7)	0.3575
TS PFAS+: TS PFAS-	1662	(617.7, 2707.1)	**0.0001**

Supplementary File 1: Scan sampling behavioral data.csv
List of behavioral states recorded from BORIS output (39516 rows).

Supplementary File 2: ViCTA behavioral data.csv
Movement counts of mosquitoes (total activity and regional activity over time, 250 rows).

Supplementary File 3: A folder containing R project to generate plots and perform statistical analysis of behavioral data
